# Cross-modular integration and the missing mechanism in biocultural accounts of language

**DOI:** 10.3389/fpsyg.2026.1765291

**Published:** 2026-09-17

**Authors:** Edward Ruoyang Shi, Ruihong Jiang, Ran Pei

**Affiliations:** 1 Center for Mental Health Education, Tianjin University of Finance and Economics Pearl River College, Tianjin, China; 2 College of General Education, Tianjin University of Finance and Economics Pearl River College, Tianjin, China; 3 Center for Mental Health Education, Tianjin Chengjian University, Tianjin, China

**Keywords:** cross-modular integration, episodic memory, event-based cognition, hippocampus, language evolution, Neo-UnCartesian linguistic program, syntax

… the totality is not, as it were, a mere heap, but the whole is something beside the parts …

— Aristotle, Metaphysics, VIII.6, translated by W. D. Ross

## 1 Introduction: from multiple facets to a unified system

Arnon et al. (2025) propose a timely and influential biocultural framework for human language, emphasizing the convergence of multiple facets—vocal learning, linguistic structure, and social cognition. Their “facets” approach reflects a growing consensus across linguistics, comparative cognition, and neuroscience that no single domain can account for the emergence of language. In this respect, the target article succeeds in consolidating decades of interdisciplinary progress and situating language evolution within a broader biocultural landscape.

However, precisely because the “many facets” view is now widely accepted, the central explanatory challenge has shifted. The crucial question is no longer which cognitive or biological systems contribute to language, but how these systems are integrated into a single, flexible, and generative capacity. Facet-based frameworks specify relevant components, yet often stop short of articulating the neural and computational mechanisms that bind them into a unified language system.

This Opinion argues that cross-modular integration, rather than mere coexistence of multiple faculties, is the key missing element in current biocultural accounts. Drawing on cognitive neuroscience, memory research, biolinguistics, and comparative event cognition, I suggest that the hippocampal episodic system provides one plausible neural component of this integrative architecture, linking sequential, syntactic, and social information within structured event representations (Duff and Brown-Schmidt, 2012; Mak et al., 2025; Rolls et al., 2025; Shi, 2024, 2025a,b, 2026a,c; Wilson et al., 2022; See also Shi, 2026b for related discussion of how Neo-UnCartesian Linguistic Program addresses this issue). This mechanistic proposal is intended to complement, rather than replace, a biocultural account: the relevant interfaces develop and evolve within culturally organized environments whose communicative practices and symbolic artifacts repeatedly reshape learning, cognition, and selection (Sinha, 2015, 2025).

## 2 The limits of facet-based explanations

Facet-based frameworks have clear strengths. They avoid reductive single-cause explanations and align well with comparative data showing partial continuities across species. Vocal learning is shared with songbirds, parrots, and cetaceans; hierarchical or combinatorial structure appears in limited form in action planning, tool use, and music; social cognition is widespread among primates and other social mammals. Arnon et al. (2025) skillfully synthesize these lines of evidence, building on broader biocultural work that links language to converging biological and cultural trajectories.

Yet enumeration alone does not explain coherence. Human language is not merely the additive result of vocal imitation, combinatorial structure, and social inference. What distinguishes language is that these elements operate in a tightly coupled and temporally extended manner. Speakers routinely refer to absent or hypothetical events, maintain reference across long discourse stretches, embed social intentions within syntactic structures, and integrate prior context into ongoing interpretation. These properties require more than the presence of multiple faculties; they require a mechanism that coordinates them in real time and over longer time scales (Duff and Brown-Schmidt, 2012).

Without an explicit integrative mechanism, facet-based models risk becoming catalogs rather than explanations. They specify what components must be present for language to exist, but remain largely silent on how these components interact during acquisition, online processing, or evolution. This limitation becomes especially salient when considering phenomena such as long-distance reference, narrative coherence, pragmatic inference, and perspective-taking, where structural relations, memory systems, and social cognition are deeply intertwined rather than In review functionally separable (Brown-Schmidt and Duff, 2016; Clough et al., 2022; Duff and Brown-Schmidt, 2012; Race et al., 2013).

From an evolutionary perspective, this gap is critical, but it cannot be formulated solely in terms of selection on an already integrated organism. In biocultural niche construction, evolving populations modify the social, material, and symbolic environments in which development and learning occur; those modified environments then alter the developmental resources and selective pressures encountered by later generations (Sinha, 2015, 2025). Language is therefore both a capacity supported by an integrated cognitive architecture and a socially inherited symbolic artifact that helps construct the niche in which that architecture is acquired and exercised. A complete biocultural theory must connect these levels: it must explain how neural and cognitive systems become coordinated, and how communicative practices, conventions, and artifacts stabilize, amplify, and diversify that coordination across ontogenetic and historical time.

## 3 Cross-modular integration as the core problem

From a cross-modular perspective, the defining feature of language is not any single faculty, but the coordination of distinct cognitive systems within a shared representational space. Syntax contributes abstract relational structure, independently of semantic content (Chomsky et al., 2019). Vocal learning supplies fine-grained sequential motor control and predictive timing mechanisms that scaffold fluent production (Gastaldon et al., 2024; Jarvis, 2019). Social cognition, in turn, provides intentions, interactional roles, and normative constraints that make linguistic acts interpretable within shared contexts (Brown-Schmidt and Duff, 2016; Levinson, 2016; Tomasello, 2010). The core theoretical problem, therefore, is how these heterogeneous representational formats are brought into alignment within a single cognitive architecture (Boeckx and Benítez-Burraco, 2014).

Recent work in cognitive neuroscience, comparative cognition, and event-cognition research converges on the idea that event representation is a phylogenetically deep foundation of cognition, including capacities for parsing agent–action–patient relations and, in some species, remembering or anticipating events beyond the immediately perceived situation. Evidence from nonhuman primates suggests that the decomposition of events into agents, actions, and patients predates human language and may have provided an important evolutionary substrate for syntax (Wilson et al., 2022). Human language, however, is not therefore merely event-based rather than symbolic. Its distinctiveness lies in the symbolic transformation of this older event architecture: linguistic systems encode event roles and inter-event relations in conventional forms that can be detached from the here and now, productively recombined, embedded, and transmitted between minds. This transformation supports elaborate mental time travel and the communication of temporally and spatially displaced events, while languages and cultures vary in how they conventionalize temporal relations and event structure (Sinha and Gärdenfors, 2014). Event representation thus supplies a prelinguistic organizational substrate; symbolic language restructures and extends that substrate rather than simply mirroring it.

This evolutionary continuity-and-transformation account has important consequences. The integrative substrate of language must preserve the relational organization of events while supporting their symbolic recoding into structures that are conventional, hierarchically organized, and available for displacement and cultural transmission. It must link abstract grammatical relations with perceptual, motor, mnemonic, and social information, while also allowing event components to be selectively foregrounded, recombined, and redescribed. These requirements point beyond a self-contained language module toward recurrent interfaces among systems for event cognition, episodic memory, syntactic structuring, action, and social inference. The hippocampal system is proposed below as one candidate contributor to this interface architecture, not as the sole source of symbolic capacity.

## 4 The hippocampal system as a cross-modular hub

A growing body of empirical and theoretical evidence points to the hippocampal system as a central candidate for this integrative role. First, episodic memory research increasingly shows that the hippocampus encodes multiplexed event structure representations—binding spatial, temporal, sequential, and contextual features into unified event models (Liu et al., 2022). Far from being static snapshots, these representations are dynamically reconstructable and updatable, allowing memory updating, recombination, and prediction (Spens and Burgess, 2024; Wahlheim and Zacks, 2025). This dynamic, event-based memory capacity mirrors the representational flexibility required for language comprehension and interpretation.

Second, recent neurocognitive studies demonstrate direct hippocampal involvement in language processing beyond lexical access. Mak et al. (2025) show that naturalistic language comprehension recruits episodic memory mechanisms, generating context-specific representations that shape semantic space over at least 12 hours. Covington and Duff (2025) similarly report that hippocampal activity supports long-term maintenance of linguistic representations, challenging traditional consolidation views that sharply separate episodic and semantic memory.

Third, formal interface work argues that hippocampal indexing mechanisms can link syntactic structures to event representations, effectively grounding abstract grammatical relations in episodic content (Shi, 2024). On this view, syntax provides a relational scaffold—defining roles, dependencies, and scopes—while episodic memory supplies temporally structured content. Language emerges from the interaction between these systems rather than from syntax alone.

Fourth, systems-level models indicate that hippocampal episodic “snapshots” train and update neocortical semantic networks through slow consolidation processes. Rolls et al. (2025) propose a biologically plausible account in which semantic attractor networks in anterior temporal cortex learn slowly from hippocampal inputs, allowing event-bound episodic representations to shape long-term semantic structure. This offers a mechanistic bridge between moment-to-moment language use and the gradual emergence of stable linguistic knowledge.

Finally, converging neurophysiological evidence links hippocampal dynamics to online language use. Direct recordings show that hippocampal theta–gamma rhythms track the integration of incoming words with stored knowledge (Piai et al., 2016), while neuroimaging suggests that hippocampal activity supports predictive processing during speech production and feedback control (van de Ven et al., 2020).

## 5 Social cognition, sequence learning, and memory

The integrative role of the hippocampus becomes even clearer when social cognition and vocal learning are considered together. Single-unit recordings in humans reveal that neurons in the hippocampus and amygdala encode social inferences in a domain-specific manner, representing not only individual identities but also relational structures such as trust, preference, and intentional stance (Cao et al., 2024). These social representations are inherently event-based and temporally organized, aligning closely with the requirements of discourse-level language use, where speakers must track who knows what, who intends what, and how these states evolve over time.

Studies of communication in amnesia provide further support. Clough et al. (2022) show that patients with bilateral hippocampal damage exhibit impairments in “audience design”—adapting utterances to a listener's knowledge state—suggesting that rich episodic reconstructions are required to imagine others' mental states and tailor language accordingly. This dovetails with earlier work linking hippocampal damage to deficits in referential processing and discourse coherence (Duff and Brown-Schmidt, 2012).

Comparative research on vocal learning highlights parallel principles. Fortkord and Veit (2025) report that social context modulates sequence modification learning in birdsong, indicating that vocal learning is embedded within social and mnemonic frameworks rather than being a purely motor skill. Although birdsong is not homologous to human language, these findings underscore a general pattern: sequence learning is shaped by memory systems and social evaluation, not isolated from them.

Event-based neuroimaging studies extend this picture. Park et al. (2025) show that hippocampal systems support encoding and sequencing of events during narrative comprehension, even when the temporal order of scenes is scrambled. Wang et al. (2025) and Liu et al. (2025) further demonstrate that hippocampal–default mode network (DMN) interactions at event boundaries influence how central and peripheral events are represented and later recalled. Kwon et al. (2025) report that coordinated hippocampal population codes support naturalistic memory retrieval, providing a dynamic substrate for constructing coherent narratives.

Taken together, these findings suggest that the hippocampal system functions as a cross-modular hub, integrating sequential structure, social meaning, and episodic context. This integration enables language to function as a tool for communicating about events rather than merely producing sequences of sounds.

## 6 Implications for biocultural theories of language

From this perspective, Arnon et al. (2025) framework provides an important inventory of interacting biological and cultural facets, but the explanatory task has two complementary levels. The first is mechanistic: how are vocal, syntactic, mnemonic, and social processes coordinated in individual brains during development and language use? The second is biocultural: how do communicative practices, conventional signs, and symbolic artifacts construct the developmental niche in which these interfaces are stabilized and modified across generations? Cross-modular integration addresses the first question, whereas niche-construction theory specifies the reciprocal organism–environment dynamics that connect ontogeny, cultural history, and biological evolution (Sinha, 2015, 2025). Neither level is sufficient by itself.

Second, it generates testable predictions. If episodic memory systems play a central integrative role, then disruptions to hippocampal function should selectively impair discourse coherence, reference tracking, and pragmatic integration—patterns observed in hippocampal amnesia, temporal lobe epilepsy, and some psychotic disorders. Existing neuropsychological work already points in this direction (Duff and Brown-Schmidt, 2012), and future studies can refine these predictions by linking specific event-segmentation deficits to linguistic symptoms. Third, this perspective aligns theories of language evolution with broader trends in evolutionary psychology that emphasize domain interaction, neural reuse, and event-based cognition. Rather than treating language as an outlier, it becomes a particularly powerful instantiation of general principles governing how brains build, update, and share structured event representations (Rolls et al., 2025).

## 7 Conclusion: toward mechanistic integration

The biocultural turn in language science has successfully moved the field beyond single-factor explanations. The next step is not simply to move from facets to mechanisms, but to connect mechanisms with the developmental and cultural niches in which they emerge. This Opinion has argued that cross-modular binding, with the hippocampal episodic system as one plausible contributor, can help explain how syntactic, mnemonic, sensorimotor, and social information is coordinated in the construction of event representations.

These event representations should not be understood as alternatives to symbols. Comparative evidence suggests that event decomposition has deep evolutionary roots, whereas human language symbolically transforms this substrate by supporting detached reference, hierarchical recombination, and culturally conventionalized relations among events. Language is consequently both an achievement of integrated cognitive architecture and a biocultural artifact that reshapes the developmental environment of subsequent learners.

Future research should therefore examine a reciprocal causal cycle: how neural systems construct and symbolically recode events; how communicative interaction and artifacts stabilize those mappings; and how culturally inherited niches feed back on development, linguistic diversity, and evolutionary change. Connecting hippocampal event representation, neocortical semantic learning, predictive production, social inference, and niche construction will bring biocultural theories closer to explaining not only what language is made of, but how its components became integrated, transformed, and diversified.
